# Management of thoracic trauma in emergency service: Analysis of 1139 cases

**DOI:** 10.12669/pjms.291.2704

**Published:** 2013

**Authors:** Isa Dongel, Abuzer Coskun, Sedat Ozbay, Mehmet Bayram, Bahri Atli

**Affiliations:** 1 Isa Dongel, Dept. of Thoracic Surgery, Suleyman Demirel University, Isparta, Turkey.; 2 Abuzer Coskun, Dept. of Emergency, Cumhuriyet University, Sivas, Turkey.; 3 Sedat Ozbay, Dept. of Emergency, Sivas Numune Hospital, Sivas, Turkey.; 4 Mehmet Bayram, Dept. of Chest Disease, Bezmialem Vakif University, Istanbul, Turkey.; 5 Bahri Atli, Dept. of Emergency, Karabuk State Hospital, Karabuk, Turkey.

**Keywords:** Thoracic trauma, Hemothorax, Pneumothorax, Rib fractures, Mortality

## Abstract

***Objective:*** Thoracic trauma is a common cause of significant morbidity and mortality. This study presents a series of thoracic trauma with the aim to assess epidemiologic features, distribution of pathologies, additional systemic injuries, diagnosis, management and outcome.

***Methodology:*** Between January 2007 and December 2011, all patients with thorax trauma admitted to the emergency service of our hospital were retrospectively reviewed with respect to age, gender, etiological factors, distribution of pathologies, additional systemic injuries, diagnosis, treatment modalities, referral and outcome.

***Results:*** A total of 1139 patients with thorax trauma were included in the study. Of these, 698 (61.3%) were male and 441 (38.7%) were female, and the average age was 54.17±17.39 years. 1090 (95.7%) of the patients had blunt trauma, whereas 49 (4.3%) had penetrating trauma. Etiological factors were falls in 792 (69.5%), motor vehicle accidents in 259 (22.8%), animal related accidents in 39 (3.4%) and penetrating injuries in 49 (4.2%) patients. It was found that 229 (20%) patients had single, 101 (8.9%) had double, 5 (3%) had three or more, 10 (0.9%) had bilateral rib fractures and 19 (1.7%) had sternal fracture. Pneumothorax was diagnosed in 58 (5.1%) patients, whereas hemothorax, hemopneuomothorax and other system injuries were diagnosed in 36 (3.2%), 38(3.3%) and 292 (25.6%) respectively. In our series, thirteen patients (mortality rate 1.1%) died as result of hemorrhagic shock (n=8), respiratory distress (n=3) and severe multiple trauma (n=2).

***Conclusion: ***Although majority of the patients with thorax trauma receive treatment as outpatients; thoracic traumas may be a life threatening condition, and should be identified and treated immediately. Mortality varies based on etiological factors, additional systemic pathologies, capabilities of the hospital especially diagnostic and treatment facilities in emergency services. We believe that a multidisciplinary approach to the patients with severe thorax trauma, and the opportunities of emergency bedside thoracotomy in emergency services will significantly reduce the morbidity and mortality.

## Introduction

 Mortality due to trauma rank third after cardiovascular diseases and cancers among the causes of adult death worldwide. Thoracic trauma constitutes 20-25% of all death due to trauma in the first four decades.^1^ As esophagus, trachea, heart, diaphragm and large vessels can be injured along with chest cage and lungs in thoracic trauma, it may be a life-threatening condition in some cases.^2^ Thus, we planned retrospective clinical study of patients with thorax trauma with the aim to assess epidemiologic features, distribution of pathologies, additional systemic injuries, diagnosis, management and outcome.

**Table-I:** Etiological reasons in thoracic traumas

		*N*	*%*
Blunt thoracic traumas	Low impact fall (while walking, stairs, etc.)	522	45.8
	High impact fall (2 m and above)	270	23.7
	Accident inside vehicle	94	8.3
	Accident outside vehicle	165	14.5
	Animal accident	39	3.4
Penetrating thoracic traumas	Stabbing injuries	32	2.8
	Firearm injuries	17	1.4
TOTAL		1139	100

## Methodology

 Records of all patients with thorax trauma admitted to the emergency service of Sivas Numune Hospital between January 2007 and December 2011 were retrospectively reviewed in terms of age, gender, distribution by seasons and years, pathologies in thorax, rates of emergent tube thoracostomy, additional systemic injuries, hospitalization time, referral to tertiary centres, and mortality rates. Pediatric patients under 16 years of age were excluded. After admission, all the patients were evaluated by baseline physical examination, plain radiography, electrocardiography and blood tests. Ultrasonography and computed tomography of thorax were used in some cases when necessary. Tube thoracostomy was performed in all patients with pneumothorax or hemopneumothorax by a thoracic surgeon either at the emergency service or thoracic surgery clinic. The patients were evaluated in terms of coexisting pathologies by the concerned traumatology physicians at the emergency service. Most of the patients with thorax trauma were hospitalized at thoracic surgery clinic, and others who were hospitalized at other clinics due to additional pathologies were followed up closely.

**Table-II:** Distribution of thoracic traumas according to seasons and years

*Year*	*N (%)*	*N (%)*	*N (%)*	*N (%)*	*TOTAL*
2007	37 (17.9)	27 (13.0)	80 (38.6)	63 (30.4)	207 (100)
2008	47 (24.0)	58 (29.6)	47 (24.0)	44 (22.4)	196 (100)
2009	86 (33.5)	57 (22.2)	42 (16.3)	72 (28.0)	257 (100)
2010	72 (27.3)	77 (29.2)	56 (21.2)	59 (22.3)	264 (100)
2011	59 (27.4)	71 (33.0)	59 (27.4)	26 (12.1)	215 (100)
TOTAL	301 (26.4)	290 (25.5)	284 (24.9)	264 (23.2)	1139 (100)

## Results

 A total of 1139 patients with thorax trauma were included in the study. Of all the patients, 698 (61.3%) were male and 441 (38.7%) were female. The majority of the patients presented with blunt thoracic trauma (1090 of 1139 [95.7%]), whereas 49 (4.3%) presented with penetrating injuries. Etiological factors included falls in 792 (69.5%) patients, motor vehicle accidents in 259 (22.8%), animal related accidents in 39 (3.4%) and penetrating injuries in 49 (4.2%). Among penetrating injuries, 32 (2.8%) were stabbing injuries and 17 (1.4%) were firearm injuries (Table-I). Distribution of the thoracic traumas by seasons and years were similar (Table-II). The patients were aged between 16 and 89 (mean 54.17±17.39) years. The vast majority (70.2%) of the patients were aged between 31 and 70 years. Blunt thoracic traumas were observed most frequently in patients aged 51-70 years (37%), whereas penetrating traumas were observed most frequently in those aged 31-50 (33,2%) years (Table-III).

**Table-III:** Distribution of thoracic traumas according to age range

	*N*	*16-30 years* *(%)*	*31-50 years* *(%)*	*51-70 years* *(%)*	*>70 years* *(%)*
Low impact fall	522	45 (8.6)	172 (33.0)	196 (37.5)	109 (20.9)
High impact fall	270	21 (7.8)	95 (35.2)	100 (37.0)	54 (20.0)
Accident inside vehicle	94	9 (9.6)	32 (34.0)	39 (41.5)	14 (14.9)
Accident outside vehicle	165	15 (9.1)	45 (27.3)	65 (39.4)	40 (24.2)
Animal accident	39	1 (2.6)	9 (23.1)	14 (35.9)	15 (38.5)
Stabbing injuries	32	10 (31.3)	16 (50.0)	6 (18.6)	-
Firearm injuries	17	4 (23.5)	9 (52.9)	1 (5.9)	3 (17.6)
TOTAL	1139	105 (9.2)	378 (33.2)	421 (37.0)	235 (20.6)

 In term of thoracic pathologies; rib and sternum fractures, pneumothorax, hemopneumothorax, and coexisting injuries were observed in the patients. Seven hundred fifty five (66.2%) patients had no fracture, whereas sternal fractures were diagnosed in 19 (1.7%), and one or more rib fractures were diagnosed in 375 (33.0%), in which 20.1% (n=229) had single, 8.9% (n=101) had two, 3.1% (n=35) had three or more, 0.9% (n=10) had bilateral rib fractures. In single rib fractures, fracture was right sided in 111 (0.9%) and left sided in 118 (10.3%). In 2 rib fractures, fractures were right sided in 43 (3.8%) and left sided in 58 (5.1%). In rib fractures more than 3, fractures were right sided in 17 (1.5%) and left sided in 18 (1.6%) (Table-IV). Pneumothorax was diagnosed in 58 (5.1%) patients (23 right sided, 34 left sided and 1 bilateral). Hemothorax was diagnosed in 36 (3.2%) patients (15 right sided, 16 left sided and 5 bilateral), and hemopneumothorax was diagnosed in 38 (3.3%) patients (15 right sided, 17 left sided and 6 bilateral). Pneumothorax was observed more frequent in blunt traumas, whereas hemothorax and hemopneumothorax were observed more frequent in penetrating traumas. Coexisting injuries were detected in 292 (25.6%) of the patients; brain injury in 52 (4.6%), abdominal injury in 95 (8.3%), and muscoskeletal injury in 145 (12.7%). The vast majority of these were related with blunt traumas (Table-V).

**Table-IV:** Rib and sterna fractures

		*No Fracture* *(%)*	*1 fracture*		*1-3 fracture*		*≥3 fracture*		*Bilateral fracture*	*Sternal fracture*
			*Right*	*Left*	*Right*	*Left*	*Right*	*Left*		
Blunt thoracic traumas	Low impact fall	471 (90)	19	21	6	5	-	-	-	-
	High impact fall	118 (43)	42	46	15	25	9	6	3	6
	Accident inside vehicle	15 (15)	23	19	6	9	4	7	2	9
	Accident outside vehicle	103 (62)	17	21	10	12	3	2	3	4
	Animal accident	8 (20)	6	7	5	7	1	3	2	-
Penetrating thoracic traumas	Stabbing injuries	26 (81)	2	3	1	-	-	-	-	-
	Firearm injuries	14 (82)	2	1	-	-	-	-	-	-
TOTAL		755 (66.2)	111 (9.7)	118 (10.3)	43 (3.7)	58 (5.0)	17 (1.4)	18 (1.5)	10 (0.8)	19 (1.6)

 Outpatient treatment, referral, hospitalization and mortality rates are shown in Table-VI. 688 (60.4%) of the cases were treated as outpatients, while 419 (36.8%) patients were hospitalized for treatment. The average length of hospitalization was 6,7 days. Emergency thoracotomy was performed in 9 patients with penetrating injury at operating room. Of these; 4 patients who had severe intercostal artery bleeding, pulmonary and diaphragm injuries were operated successfully, unfortunately 5 died duo to major vascular and cardiac injuries during surgery. Nineteen patients (1.7%) were referred to a tertiary centre because of deterioration in general condition and coexisting pathologies, including spinal injury due to vertebral fracture (n=5), subdural haemorrhage and cerebral contusion (n=4), hemorrhagic shock due to laceration of liver and/or spleen (n=3), requirement for dialysis due to CRASH syndrome (n=1), respiratory failure (n=2), hemorrhagic shock due to cardiac and main vascular injuries (n=4). In our case series, mortality rate was 1.1% (n=13). Cause of mortality were hemorrhagic shock (n=8), respiratory failure (n=3), cardiac contusion (n=1) and respiratory arrest due to dislocation of upper cervical vertebra (n=1). All of these were intubated in emergency service, and cardiopulmonary resuscitation was performed. Blood transfusion was also carried out in patients in hemorrhagic shock.

**Table-V:** Pneumothorax, hemothorax, hemopneumothorax due to thoracic traumas and accompanying

		*Pneumothorax*			*Hemothorax*			*Hemopneumothorax*			*Brain*	*Abdominal*	*Musco-skeletal*
		*Right*	*Left*	*Bilateral*	*Right*	*Left*	*Bilateral*	*Right*	*Left*	*Bilateral*			
Blunt thoracic traumas	Low impact fall	1	-	-	-	-	-	-	-	-	4	7	12
	High impact fall	4	6	-	2	3	2	2	3	1	16	27	64
	Accident inside vehicle	8	12	1	2	1	1	-	-	1	19	23	24
	Accident outside vehicle	3	2	-	-	1	-	1	1	-	7	11	13
	Animal accident	2	7	-	2	2	1	1	2	1	1	9	11
Penetrating thoracic traumas	Stabbing injuries	3	6	-	6	7	1	6	4	1	3	13	12
	Firearm injuries	2	1	-	3	2	-	5	7	2	2	5	9
TOTAL		23	34	1	15	16	5	15	17	6	52	95	145

^#^ Tube thoracostomy was performed in all the patients undergoing pneumothorax or hemopneumothorax due to traumatologic reasons.

**Table-VI:** Outpatient treatment, referral, hospitalization and mortality rates in thoracic traumas

	*Outpatient treatment (%)*	* Hospitalization* * (%)*	*Referral* * (%)*	*Mortality* *(%)*
Low impact fall	449 (86.0)	72 (13.8)	1 (0.2)	**––**
High impact fall	130 (48.4)	136 (50.5)	3 (0.01)	1 (0.003)
Accident inside vehicle	24 (25.5)	63 (67)	4 (4.3)	3 (3.2)
Accident outside vehicle	72 (43.9)	89 (54)	3 (0.02)	1 (0.01)
Animal accident	11 (28.2)	25 (64.1)	2 (5.1)	1 (2.6)
Stabbing injuries	2 (6.3)	23 (71.8)	4 (12.5)	3 (9.4)
Firearm injuries	**––––**	11 (64.7)	2 (11.8)	4 (23.5)
TOTAL	688 (60.4)	419 (36.8)	19(1.7)	13 (1.1)

## Discussion

 Thoracic injuries mostly occur as a component of multiple traumas. Early diagnosis and treatment is life-saving for acutely injured patients. The management principles are simple and straightforward. The best management for these patients includes early mobilization, aggressive pain control, proper fluid management, and respiratory physiotherapy. Endotracheal intubation should be reserved for the patients with airway compromise, refractory problems with gas exchange, hypoventilation, and decreased mental status. Prophylactic intubation is not a desired measure for severe chest wall injury.^3^ The elderly patients carry the greatest risk for pneumonia, respiratory failure, and multiple organ failure. Special efforts at aggressive regional pain control are probably beneficial in this group of high risk patients.^4^

 The most complications in these patients include respiratory failure due to altered chest wall mechanics from the fractures and respiratory distress from fracture-associated pain. Underlying pulmonary contusion plays a prominent role in the hypoxia that develops after chest wall injury. This complex pathophysiology often necessitates endotracheal intubation, prolonged mechanical ventilation, tracheostomy, and prolonged intensive care unit length of stay.^5^ In addition, poor pulmonary function and mechanical ventilation increase the risk for the development of pneumonia, which is a frequent cause of death.^6^ Several factors such as age, the total number of fractures, and the presence of bilateral fractures have been shown to contribute to the morbidity and mortality associated with thoracic wall injury. 

 Thoracic trauma constitutes majority of the trauma cases in emergency clinics. One third of the hospitalizations for trauma consist of thoracic injuries. In this respect, the results of the present study are similar with the literature. The frequencies of blunt trauma and penetrating injuries have been reported as 58-75% and 24-41% respectively.^1^^,^^2^ Another study from Canada reported frequency of blunt thoracic trauma as 96.3%.^7^ In our study, 95.7% of the patients had blunt and 4.3% had penetrating thoracic trauma, which was similar to the results obtained in the Canadian study. We believe that different results are associated with different socio-economical status, developmental levels and opportunities in emergency clinics. Low ratio of penetrating injury in our study may be related to the facts that some of the patients with penetrating injury were taken to a third level hospital or some lost their lives before taken to a hospital. Distribution of the thoracic traumas by seasons and years were similar in our study. The vast majority (70.2%) of the patients were aged between 31 and 70 years. Blunt thoracic traumas were observed most frequently in patients aged 51-70 years, whereas penetrating traumas were observed most frequently in those aged 31-50 years. Motor vehicle accidents and falls are the most frequently observed etiological factors in thoracic traumas.^8^ Motor vehicle accidents have been reported as the most common etiological factor with a frequency between 42% and 80.2% in large series.^9^^,^^10^ In our study, falls were the most frequent etiological factor with a ratio of 69.5%, and falls were followed by motor vehicle accidents (26.2%). We believe that different ratios in our study were caused by the fact that the study region has a cold climate, thus falls while walking or from height were observed frequently due to snow and ice. 

 Postero-anterior lung X-ray is the most valuable diagnosis method in thoracic traumas. Routine postero-anterior lung X-ray is sufficient to diagnose rib fractures, pneumothorax, hemothorax and lung contusions. Trupka et al.^11^ reported that computed tomography is superior to posterior-anterior lung X-rays in imaging contusion, pneumothorax and hemothorax screening in blunt thoracic trauma and that it should be the first method to be used in those having multiple injuries and suspected to have thoracic trauma. We believe that postero-anterior X-ray should be performed in patients having thoracic trauma and computed tomography should be used if further examination is needed.

 Clinical features in thoracic trauma varies from a simple soft tissue injury to a life-threatening condition. Pnemothorax, hemothorax, hemopneumothorax, pulmonary contusion and rib fractures are the most frequently observed findings. Fractures occur frequently due to blunt thoracic traumas.^1^^,^^2^ In our study, one or more rib fractures were diagnosed in 33.0% of the patients, and almost all of the rib fractures occurred due to blunt thoracic trauma. Sternal fracture is observed in 3-8% of the cases with blunt thoracic trauma^12^, whereas 1.7% of the patients had sternal fracture in the present study. It is known that 18-62% of sternal fractures are accompanied with cardiac injury.^13^ We think that one of the reasons of sudden deaths at early stage is cardiac contusion, as one of the patient died due to cardiac contusion in our series. Therefore, electrocardiography and cardiac enzyme panel was carried out in all the patients suspected to have sternal fracture. The most frequently observed intrathoracic pathologies in thoracic trauma are pneumothorax, hemothorax or both, and the first stage of treatment is tube thoracostomy.^14^^,^^15^ In our study, pneumothorax was diagnosed in 5.1% of patients, whereas hemothorax and hemopneumothorax were diagnosed in 3.1% and 3.3% respectively. All of these patients were subjected to tube thoracotomy. There are publications reporting that life-threatening complications such as tension pneumothorax could be prevented by this way.^15^ On the other hand, Menger et al.^16^ reported the ratio of tube complications as 20%. We advocate that chest tube should be inserted in all pneumothorax and hemopneumothorax cases by experienced physicians. In our study, 0.8% (n=9) of the patients underwent emergency thoracotomy. The indications were hemorrhagic shock, and hemorrhagic drainage greater than 1500 cc following tube insertion, or bleeding >100 cc/hr within 6-8 hours, or >200 cc/hr within 3-4 hours.

 The most important factors affecting mortality in thoracic trauma is coexisting injuries in other systems and organs. In a study including 3406 cases, Regel et al.^17^ reported that thoracic traumas were most frequently accompanied by extremity fractures and this is followed by brain injuries. In similar studies, thoracic traumas have been reported to be accompanied most frequently by musculoskeletal injuries.^1^^,^^10^^,^^18^ In our study, 25.6% of the patients had coexisting systemic injuries, in which the musculoskeletal injuries were the most frequently observed one. In thoracic trauma, physicians in emergency service should be alert for coexisting systemic injuries.

 In our study, 60.4% of the cases admitted to the emergency service were treated as outpatients, and 36.8% were hospitalized for treatment. About 1.7% of the patients were referred to a tertiary level centre because of coexisting systemic pathologies and deterioration in their general condition. Emircan et al.^19^ studied factors affecting mortality of patients with thoracic trauma, and reported that Trauma Revised Score-Injury Severity Score has been the strongest factor in determining mortality and thus patients with thoracic trauma should be treated as a high risk group and diagnosis and treatment should be aggressive. Although our approach towards the patients with thoracic trauma was aggressive too, early stage mortality was observed in 1.1% of the patients. In some studies, mortality rates have been found to be increased in patients aged 45 and less, and 65 and over. Mortality rates have been found to be significantly higher in penetrating injuries and traffic related injuries to pedestrians when compared to other injuries. Akcam et al.^20^ performed emergency bedside thoracotomy in 6 patients with penetrating injury and 3 patients with blunt thoracic trauma, and success was only achieved in 3 patients with penetrating injury. They concluded that emergency bedside thoracotomy is a life-saving procedure in the cases of penetrating thoracic injury. As we had no facility of emergency bedside thoracotomy in emergency services, thoracotomies were compulsorily performed in operating room. We lost minutes, which is important for the patients. Emergency thoracotomy was performed in 9 patients with penetrating injury. Of these, 4 were operated successfully. Unfortunately, 5 patients died due to major vascular and cardiac injuries during the operation. We think that emergency bedside thoracotomy may be a life-saving procedure in these patients.

 In conclusion, although some of the patients with thorax trauma receive treatment as outpatients; thoracic traumas may be a life threatening condition, and should be identified and treated immediately. Mortality varies based on etiological factors, additional systemic pathologies, capabilities of the hospital especially diagnostic and treatment facilities in emergency services. We believe that a multidisciplinary approach to the patients with severe thorax trauma, and the opportunity of emergency bedside thoracotomy in emergency services will significantly reduce the morbidity and mortality.
